# Retention of the Pre-Descemet’s Layer Reduces Endothelial Graft Scrolling: Digital Morphometric Analysis of Precut Endothelial Grafts

**DOI:** 10.3390/diagnostics16111660

**Published:** 2026-05-28

**Authors:** Rodrigo B. Prado, Sergio M. Borghi, Ana Paula M. T. Oguido, Waldiceu A. Verri, Antônio Marcelo B. Casella

**Affiliations:** 1 Surgical Clinic Department, Universidade Estadual de Londrina, Londrina 86057-970, Brazil; 2 Pathological Science Department, Universidade Estadual de Londrina, Londrina 86057-970, Brazil

**Keywords:** endothelial keratoplasty, eye bank, trypan blue, Pre-Descemet endothelial keratoplasty (PDEK), Descemet membrane endothelial keratoplasty (DMEK)

## Abstract

**Purpose**: To evaluate the biomechanical impact of the Pre-Descemet’s Layer (PDL) on central graft width (CGW) and Scrolling Score (SS) using a standardized digital morphometric approach in pre-Descemet endothelial keratoplasty (PDEK) and Descemet membrane endothelial keratoplasty (DMEK) grafts. **Methods**: This experimental study analyzed 47 donor corneoscleral buttons. Grafts were prepared via pneumatic dissection into PDEK or DMEK. CGW and SS were quantified using FIJI-ImageJ software from standardized video frames. Statistical analysis included Student’s *t*-tests, Pearson’s correlation, and Chi-Square tests. Endothelial viability and global integrity were assessed in representative PDEK grafts using confocal microscopy with triple vital staining (Hoechst 33342, Ethidium-homodimer, and Calcein-AM). **Results**: Forty-one grafts were successfully prepared (24 PDEK; 17 DMEK). The failure rate was significantly higher in diabetic donors (50% vs. 7.3% in non-diabetics, *p* < 0.001). PDEK grafts exhibited a significantly higher mean CGW (2.35 ± 0.36 mm) compared to DMEK (1.95 ± 0.41 mm; *p* < 0.05). A clear inverse relationship was established between CGW and SS; SS-2 was predominant in PDEK (50%), while SS-3 prevailed in DMEK (70.5%). Donor age showed a weak correlation with CGW in DMEK (R^2^ = 0.283) and no correlation in PDEK (R^2^ = 0.0004). Trypan blue staining did not significantly alter CGW or SS in either group (*p* > 0.05). Confocal microscopy confirmed high endothelial viability (2407 cells/mm^2^) with minimal iatrogenic damage in tissue folds. **Conclusions**: Retention of the PDL significantly reduces endothelial graft scrolling and increases CGW values in PDEK compared with DMEK. Although donor age demonstrated a weak association with CGW in DMEK grafts, no such relationship was observed in PDEK. Digital morphometric analysis represents a reproducible approach for preoperative characterization of graft behavior in eye banks.

## 1. Introduction

Precut corneas processed by the eye bank facilitate the distribution of dissected grafts, reducing the surgical complexity of posterior lamellar corneal transplantation [1,2].When submerged, an endothelial graft spontaneously forms a roll with the endothelium facing outwards [3]. This scrolling tendency is attributed to the high elastin content in the anterior fetal portion of Descemet’s membrane (DM) [4]. As the posterior portion of the DM thickens progressively with age due to continuous endothelial cell secretions, surgeons typically prefer donors older than 50 years for Descemet’s membrane endothelial keratoplasty (DMEK) [5]. However, donor age and endothelial cell density (ECD) are inversely proportional [6]. While younger donor corneas offer the high ECD desirable for keratoplasty, their pronounced scrolling tendency presents significant technical challenges during the procedure [7]. The clinical significance of this phenomenon was recently highlighted by Voulgari et al. (2026), who analyzed the scrolling behavior in a large cohort of over 3000 DMEK grafts, emphasizing its impact on surgical outcomes [8].

A significant paradigm shift in corneal anatomy occurred in 2013 when Dua et al. identified the pre-Descemet layer (PDL), also known as Dua’s layer. This well-defined, acellular structure, measuring approximately 10 to 15 μm in thickness, is situated between the posterior stroma and DM [9]. Composed of 5 to 8 thin, ocularly strong lamellae of collagen bundles, the PDL exhibits remarkable biomechanical strength and is notably impervious to air [10]. This physical property facilitates the pneumatic dissection of endothelial grafts via the ‘big bubble’ (BB) technique across all age groups, including infant donors [11]. Consequently, PDEK emerged as a robust alternative to DMEK. By including the PDL, PDEK grafts are structurally thicker and exhibit significantly less scrolling tendency than DM-only grafts [12]. This increased rigidity results in ‘looser’ scrolls that are easier to manipulate and center within the anterior chamber. Most importantly, the use of PDL effectively expands the donor pool by allowing the successful use of young donor corneas, which provide exceptionally high ECDs that would otherwise be technically prohibitive in conventional DMEK due to excessive tissue scrolling.

To ensure intraoperative visualization and prevent primary graft failure due to inverted tissue orientation, the application of trypan blue (TB) dye has become a standard practice among surgeons and eye bank technicians. Although TB is characterized by low cellular toxicity, its role extends beyond simple staining; it remains detectable within the tissue even after prolonged storage, with the literature reporting an endothelial cell loss of only 16 to 18% in pre-stained, preloaded tissues shipped internationally [13,14]. Recent biomechanical investigations using atomic force microscopy have demonstrated that TB staining significantly increases tissue stiffness [15]. However, a critical gap remains in the literature regarding how this increased rigidity—combined with the inherent properties of the graft—affects the precise morphology and dimensions of the scrolled tissue.

Two key variables have been proposed to characterize the endothelial graft roll: the central graft width (CGW), which quantifies the lateral extent of the parchment roll [6], and the Scrolling Score (SS) [16]. The SS is a qualitative framework that classifies the morphology of the scrolled graft into four distinct stages, reflecting the tissue’s tendency to form a tight or loose rolls. Furthermore, the technical process of loading these tissues into injector cartridges requires fluid-mediated aspiration, yet discussions regarding the ideal correlation between cartridge tip diameter, corneal incision size, and graft type remain scarce. While a general consensus exists that PDEK and DMEK grafts exhibit different scrolling behaviors, these differences lack formal quantification. Consequently, this study aims to evaluate and compare the morphological characteristics of DMEK and PDEK grafts, utilizing CGW and SS as metrics. Additionally, we provide a viability analysis of a representative PDEK graft through confocal microscopy using vital dyes to assess the integrity of the endothelial layer following these manipulations.

## 2. Materials and Methods

### 2.1. Study Design and Tissue Selection

The Institutional Ethics Committee of Londrina University Hospital, Brazil, approved this study protocol under number 88172518.2.0000.5231, and we obtained informed consent during organ donation. Between September 2018 and August 2020, a single experienced surgeon (Oguido APM) prepared all donor tissues. The local eye bank provided forty-nine corneoscleral buttons originally intended for disposal. Inclusion criteria required an ECD exceeding 2000 cells/mm^2^, as measured by specular microscopy (Konam, Nishinomiya, Japan). We excluded donors with a history of glaucoma, intraocular surgery, or uveitis. The data obtained regarding the donor’s age, sex, donor diabetes mellitus, death-to-preservation time (DPT), total preservation time (TPT), procedure time (PT), and air injected volume (AIV) were documented; statistical analysis was performed using IBM-SPSS Statistics Base, version 20.0.

We stratified the study into two groups based on the type of BB formed during air injection: the PDEK group (type-1 and type-3 BB), and the DMEK group (type-2 BB). We quantified and analyzed dissection failures and associated risk factors. For each graft, we selected four video frames and performed digital measurements using FIJI-ImageJ software (version 1.53, National Institutes of Health, Bethesda, MD, USA). To calibrate the scale, we placed a 3 mm caliper in the corneal plane. We calculated the mean of three independent measurements for the following variables: CGW, posterior white-to-white distance (PWTWD), BB diameter (BBD), graft length (GL), graft area (GA), and graft margin circularity (GMC).

### 2.2. Morphological Classification (Scrolling Score)

A single masked evaluator (Prado RB) analyzed the scrolling behavior of endothelial grafts while submerged in balanced salt solution. Scrolling was classified according to the Scrolling Score (SS), a four-grade system previously described [16]. Briefly, SS-1 indicates no contact between graft edges; SS-2 indicates initial edge contact without complete scrolling; SS-3 corresponds to the formation of a single scroll; and SS-4 denotes the presence of multiple scrolls or a tightly rolled configuration. When necessary, dynamic assessment of the scrolling process was performed using recorded video frames.

### 2.3. Graft Preparation and Staining

We placed donor corneas on a trepanation block with the endothelial side upward and applied 0.1% TB (Ophthalmos, São Paulo, Brazil) for 30 s. Using a 5 cm^3^ syringe with a 30 gauge needle, we injected air into Schwalbe’s line (bevel up) until reaching the central and posterior stroma. Following BB formation, we completed the dissection using a 15-degree scalpel and scissors for PDEK grafts, or an 8.25 mm trephine (Katena, Denville, NJ, USA) for DMEK grafts. We performed a 3 mm punch on the anterior surface and applied an “S” stamp for orientation. We performed digital analysis of the endothelial graft roll submerged in BSS both before and after a 3 min TB staining period. Finally, we opened the graft roll on a filter paper using a blunt spatula for further assessment.

### 2.4. Statistical Analysis

We expressed continuous variables using descriptive statistics and counting data via total and percentage frequencies. After verifying residue normality with the Shapiro–Wilk test, we applied Student’s *t*-test to paired samples of CGW following TB staining. We utilized Pearson’s linear correlation (R) and dispersion values to examine the relationship between donor age and CGW in both groups. For unpaired samples, we used the Mann–Whitney U test, while the Chi-square test assessed the association between failed dissections and risk factors. We set statistical significance at *p* < 0.05.

### 2.5. Confocal Microscopy and Viability Analysis

To evaluate cellular integrity, we analyzed a representative PDEK graft using a Leica TCS SP8 confocal microscope (Wetzlar, Germany) attached to LAS software (Leica Microsystems, Wetzlar, Germany). We performed triple staining using 100 µL solution containing: 60 µL BSS, 30 µL Hoechst 33342 (diluted 1:5000), 10 µL Ethidium-homodimer (EH-1) and 10 µL of Calcein AM (CAM) [17]. Following a 45 min incubation and BSS rinse, we positioned the graft (endothelium-up) on a viscoelastic-coated Petri dish and flattened it with a coverslip. We captured images at 20× magnification for central areas and 5× for global mapping (a merge of nine juxtaposed images). We analyzed fluorescence across three laser channels: blue (Hoechst; viable nuclei), red (EH-1; damaged membranes), and green (CAM, viable cytoplasm).

## 3. Results

### 3.1. Donor Demographics and Baseline Characteristics

A total of 47 corneas from 27 donors were included in this study (21 bilateral and 7 unilateral cases). Two corneas from a single donor were excluded due to previous ocular surgery. The mean donor age was 53.8 ± 14.5 years (range: 18–70), with a predominance of male donors (66.6%). The mean endothelial cell density (ECD) was 2376 ± 244 cells/mm^2^. Diabetic donors accounted for 14.8% of the cohort. Detailed demographic and eye-bank records are summarized in Table 1.

### 3.2. Graft Preparation and Big Bubble Formation

The PDEK group comprised 24 corneas (51%) with type-1 BB (79.1%) and type-3 BB in 20.8% of cases. The mean air injection volume (AIV) for PDEK was 3.5 ± 3.0 cm^3^, with a preparation time (PT) of 12.5 ± 5.7 min. In contrast, the DMEK group (17 corneas, 36.2%) with type-2 BB formation had an AIV of 4.6 ± 4.1 cm^3^ and a significantly shorter PT of 4.7 ± 4.1 min.

**Figure 1:**
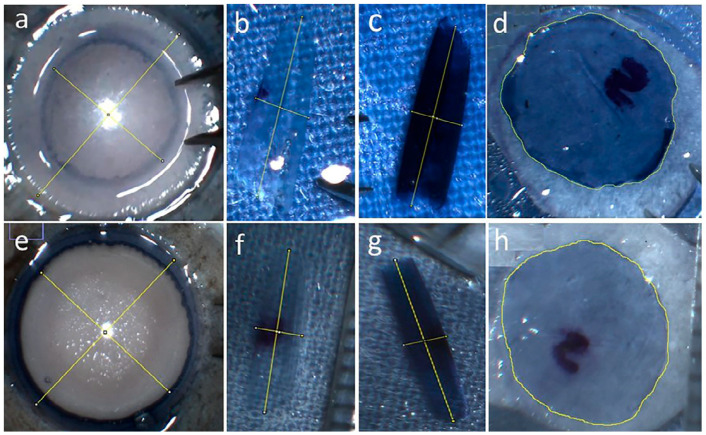
Representative frames of graft dissection for pre-Descemet endothelial keratoplasty (**upper panels**) and Descemet membrane endothelial keratoplasty (**lower panels**). Measurements were obtained using FIJI-ImageJ software: posterior white-to-white distance (PWTWD; right diagonal; (**a**,**e**)); big bubble diameter (BBD; left diagonal; (**a**,**e**)); graft length (GL; vertical line; (**b**,**c**,**f**,**g**)); central graft width (CGW; horizontal line; (**b**,**f**)); CGW after trypan blue staining (TB-CGW; horizontal line; (**c**,**g**)); graft area (GA; (**d**,**h**)); and graft margin circularity (GMC; (**d**,**h**)).

Notably, BB rupture occurred in six corneas (12.7%) from six different donors (mean AIV: 4.5 cm^3^). In all instances of failure, the contralateral corneas were successfully dissected. Digital morphometric measurements were obtained from the surgical videos using FIJI-ImageJ software (version 1.53 National Institutes of Health, USA) (Figure 1).

### 3.3. Tissue Morphometric and Trypan Blue Staining

CGW and SS did not change significantly after TB staining in either group (*p* > 0.05). In the PDEK group, grafts predominantly exhibited SS-2 (50%) and SS-3 (30%), with a mean CGW of 2.35 ± 0.36 mm. In contrast, DMEK grafts most frequently showed SS-3 (70.5%) and SS-4 (17.6%), with a significantly smaller mean CGW of 1.95 ± 0.41 mm (*p* < 0.001). A detailed analysis of the relationship between SS, CGW, and TB staining is presented in Table 2.

**Figure 2:**
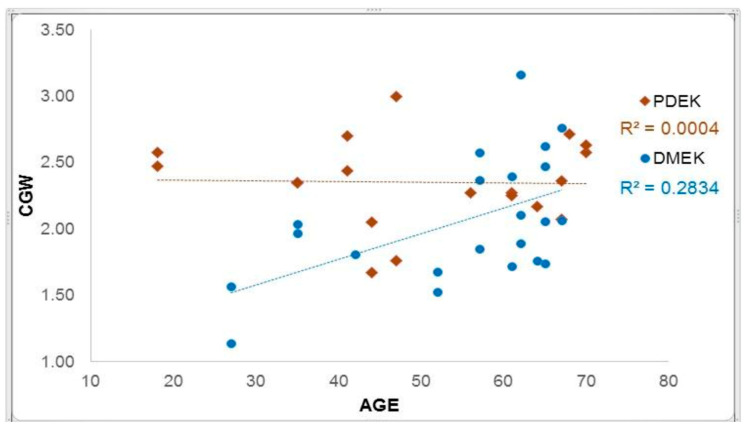
Dispersion values, determination coefficient, and Pearson linear correlation of central graft width (CGW) and age for pre-Descemet’s endothelial keratoplasty (PDEK) and Descemet’s membrane endothelial keratoplasty (DMEK) grafts.

A strong inverse relationship was observed between CGW and SS in both groups. Correlation analysis revealed that donor age had a moderate positive linear correlation with CGW in the DMEK group (R = 0.53), whereas no correlation was found in the PDEK group (R = 0.02, Figure 2).

Significant differences were identified between PDEK and DMEK regarding BBD, GL, and GA (*p* < 0.001), while PWTWD remained similar between groups (*p* = 0.496, Table 3). Furthermore, a strong association was found between failed dissection and donor diabetes mellitus; the failure rate was 50% in diabetic donors compared to only 7.3% in non-diabetic donors (χ^2^ test, *p* < 0.001).

### 3.4. Endothelial Viability Assessment

**Figure 3:**
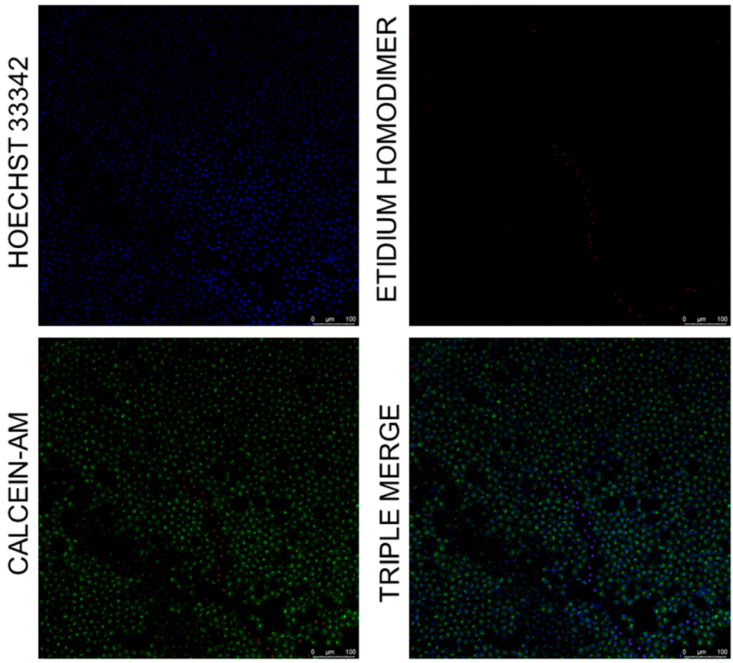
Pre-Descemet endothelial keratoplasty graft with 20× objective lens of central area and confocal microscope fluorescence analysis with triple staining. Viable endothelial cell nuclei stained with Hoechst 33342; unviable endothelial cell stained with Ethidium-homodimer; green fluorescence of live cell cytoplasm stained with Calcein AM; three laser channel overlapping images.

**Figure 4:**
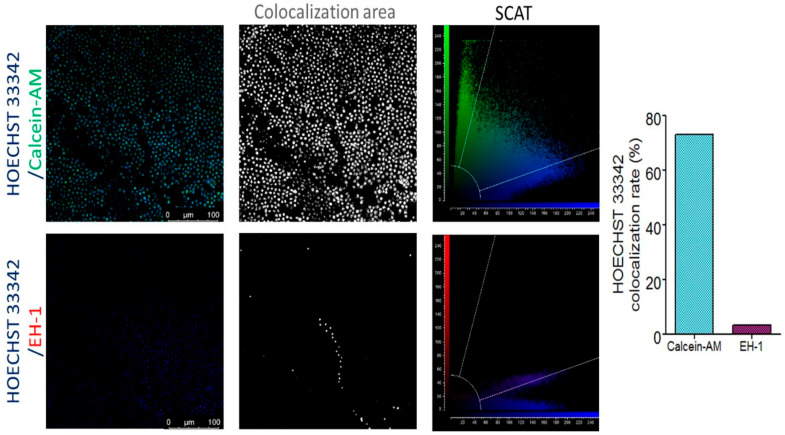
Pre-Descemet endothelial keratoplasty graft and confocal microscope fluorescence analysis. FIJI-ImageJ-processed images with percentage bar graph of Hoechst 33342-stained cells and colocalization rate with Calcein-AM or Ethidium-homodimer.

**Figure 5:**
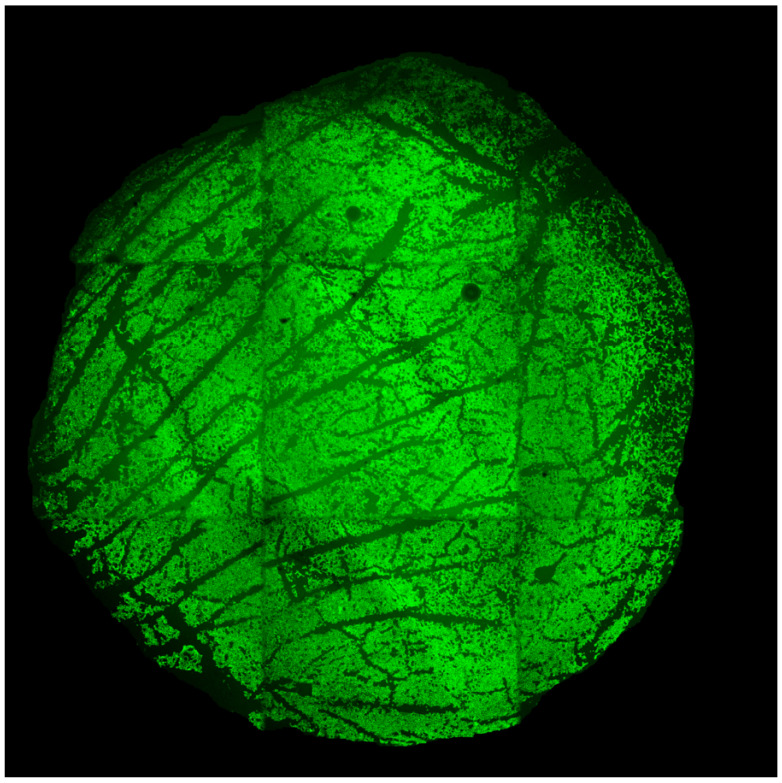
Global graft viability assessment of a representative pre-Descemet endothelial keratoplasty graft digitally mounted with nine juxtaposed images. Green fluorescence represents viable cell cytoplasm and dark areas are indicative of cellular dropout.

Confocal microscopy using vital dyes confirmed high cellular integrity in representative PDEK grafts. Viable nuclei (Hoechst positive) showed a density of 2407 cells/mm^2^, with a minimal non-viable cell count (EH-1 positive) of 37 cells/mm^2^ (Figure 3). High colocalization was observed between the green cytoplasmic fluorescence (CAM) and nuclear staining (Figure 4). A digital wide-field montage of nine juxtaposed images confirmed global graft viability, showing a confluent endothelial layer with localized dropout areas (Figure 5).

## 4. Discussion

Endothelial grafts have established themselves as the gold standard for posterior lamellar keratoplasty, offering an anatomically precise approach to corneal replacement. In this evolving landscape, the role of eye-bank tissue processing has become increasingly critical for surgical success [18,19]. The present study underscores the clinical relevance of CGW and SS as two pivotal variables characterizing graft morphology. Notably, this is the first study to implement a standardized, semi-automated approach using FIJI-ImageJ software to provide a reproducible characterization of both PDEK and DMEK grafts. These metrics serve as a proxy for the internal elastic forces acting within the tissue roll, offering the transplant team predictive insights into surgical complexity and procedure time.

Our findings suggest that ‘tight’ grafts—defined by a CGW less than 1.5 mm or SS-4—necessitate significantly more intraocular manipulation. This is a concerning factor, as prolonged operative duration and excessive surgical handling have been strongly correlated with accelerated endothelial cell loss and poor long-term graft survival [20]. Conversely, ‘loose’ grafts (CGW > 2.5 mm or SS-1) may facilitate easier intraocular unfolding; however, their larger diameter when preloaded may increase contact with the cartridge wall during injection, potentially inducing cell trauma. A further limitation to consider is the inherent biological variability in graft preparation, even when using paired corneas from the same donor. Despite their shared genetic background, the pneumodissection process can yield different bubble types or scrolling patterns in contralateral eyes, effectively resulting in distinct mechanical behaviors for each tissue. This underscores that stromal adhesion and lamellar cleavage are not always symmetrical. Some limitations of this study include the relatively small sample size, the use of corneas ineligible for transplantation due to tissue scarcity in Brazil, and the fact that endothelial cell density (ECD) was only assessed before the procedure. Therefore, the specific impact of eye-bank processing and subsequent graft manipulation on acute endothelial damage remains to be quantified in future prospective trials with larger cohorts.

The utilization of PDEK grafts processed by eye banks emerges as a promising advancement in posterior lamellar surgery, primarily due to the ability to harness tissues from younger donors who typically possess superior ECD. Our analysis revealed a statistically significant disparity in both CGW and SS between PDEK and DMEK, highlighting fundamental biomechanical differences. While any endothelial graft submerged in BSS inherently scrolls with the endothelial layer facing outwards, this phenomenon was notably less pronounced in PDEK tissues. Quantitatively, this study demonstrated that PDEK grafts exhibited a mean CGW approximately 20% greater than their DMEK counterparts. This increased width is largely attributable to the inclusion of the PDL, which effectively increases the overall thickness of the posterior lamella. The additional structural support provided by the PDL appears to counteract and attenuate the scrolling forces traditionally induced by the anterior (fetal) band of the DM, a region characterized by a dense concentration of elastin [21].

Furthermore, the predominance of SS-2 (50%) in our PDEK cohort characterizes these grafts as ‘loose’ rolls, frequently manifesting in a double-roll configuration that is clinically easier to manipulate. This observation aligns with the physiological maturation of the graft, while the constant secretion by endothelial cells throughout life progressively thickens the posterior band of the DM—thereby reducing the scrolling tendency in elderly DMEK donors [5]. The PDEK technique achieves a similar (or superior) reduction in scrolling intensity even in young tissues by incorporating the PDL. Thus, PDEK effectively bypasses the ‘tight scroll’ challenges typically associated with young donor tissue in conventional DMEK, potentially expanding the pool of viable grafts for complex reconstructive cases.

Regarding the biochemical influence on graft behavior, previous studies utilizing atomic force microscopy have suggested an increase in the stiffness of DM fragments following exposure to TB [15]. This phenomenon is hypothesized to be analogous to collagen cross-linking induced by photosensitizing agents and light energy. However, in our analysis, no statistically significant differences were observed between the CGW and TB-CGW (*p* > 0.05). Consequently, our data do not support the hypothesis that routine clinical staining significantly alters the macro-morphological rolling characteristics of the graft. A potential confounding factor in this observation is the lack of controlled exposure to light energy from the surgical microscope or operating room illumination, which may be necessary to catalyze the stiffening effect associated with TB.

In addition to staining, the application of an S-stamp to the anterior surface of the posterior lamella is a critical maneuver to ensure correct graft orientation and prevent inverted intraocular implantation—a primary cause of early graft failure in endothelial keratoplasty. In DMEK, the stamp is applied directly to the anterior face of the DM, which lies in close anatomical proximity to the endothelial layer; previous reports have identified localized endothelial cell loss in areas adjacent to these markings [22]. In contrast, the PDEK graft architecture includes the PDL, meaning the stamp is applied to the anterior surface of the PDL rather than the DM itself. Notably, our qualitative assessment of representative PDEK grafts in this study revealed no observable endothelial cell loss directly subjacent to the S-stamp marking. This suggests that the increased anatomical distance and the structural buffering provided by the PDL may offer a protective effect against iatrogenic endothelial damage during graft preparation. While these preliminary findings are encouraging, this potential advantage of PDEK warrants further systematic investigation through large-scale cohorts using specular microscopy or viability staining to definitively quantify the degree of endothelial preservation.

While young donors with high ECD are highly desirable for endothelial keratoplasty, their pronounced scrolling tendency often complicates intraocular unfolding and adherence to the recipient stroma. In the present study, both PDEK and DMEK grafts obtained from donors under 50 years of age via air dissection frequently exhibited a CGW of 1.1 mm and SS-4. These metrics characterize extremely tight grafts, which could present significant surgical challenges and, according to our findings, should ideally be flagged by eye banks as high-complexity tissues. The inherent air impermeability of the PDL serves as a fundamental anatomical landmark for successful PDEK graft preparation. Specifically, the presence of small peripheral fenestrations within this layer has been identified as a key factor facilitating the formation of type-2 BBs during air injection [23]. In routine eye bank processing, specialized rounded clamps are frequently employed to mitigate peripheral air escape and ensure the consistent formation of type-1 BBs, thereby standardizing the PDEK output. However, in the present study, we deliberately abstained from using such clamping devices. This methodological approach facilitated the natural occurrence of type-1, -2, and -3 BBs, ensuring the necessary diversity of graft types for a robust comparison between PDEK and DMEK. Nevertheless, the uneven distribution of grafts between groups constitutes a limitation of this study.

The presence of the PDL inherently stabilizes the scrolling tendency; however, the significantly higher CGW values observed in PDEK grafts necessitate a critical re-evaluation of the injector cartridge dimensions and the corresponding corneal incision width. Current surgical protocols often rely on the adaptation of intraocular lens cartridges, ranging from 2.2 mm to 3.0 mm. However, a standardized consensus for this pivotal stage of endothelial keratoplasty is currently lacking. Frequently, surgeons are compelled to manually enlarge the cartridge tip to accommodate the graft, aiming to prevent iatrogenic endothelial trauma. This is particularly relevant as the rolled tissue must navigate this physical bottleneck twice: first during aspiration into the device and subsequently during its injection into the anterior chamber. Our data suggests that eye bank processing could seamlessly integrate CGW and SS measurements into their standard reports using a single calibrated photograph. The adoption of this practice is especially pertinent for PDEK grafts, as it empowers the surgical team to preoperatively plan the ideal cartridge and incision dimensions. While our total sample yielded an average CGW of 2.4 mm, it is noteworthy that SS-1 grafts—even from younger donors—resulted in a CGW of 2.8 mm. Without prior knowledge of these dimensions, a surgeon might attempt to force a 2.8 mm graft through a standard 2.2 mm orifice, inevitably leading to significant endothelial cell loss due to excessive friction and compression.

The tight peripheral adhesion of the PDL to the corneal stroma inherently constrains the expansion of type-1 BBs. In our protocol, the meticulous use of surgical scissors was aimed at maximizing the usable diameter of the PDEK graft. When evaluating the GMC variable—where a value of 1.0 represents a perfect circle—trephined DMEK grafts achieved a mean value of 0.89, significantly higher than the 0.81 observed in PDEK grafts (*p* < 0.001). Although PDEK grafts demonstrated slightly lower circularity, a GMC of 0.81 remains within an acceptable clinical range. We argue that this minor deviation in roundness should not adversely affect clinical outcomes, as GA and ECD remain the primary predictors of the total transplanted cell count and, consequently, the successful restoration of corneal transparency [24].

Furthermore, this study offers a critical re-examination of the prior literature [6] which suggested that trephination size is equivalent to GL in DMEK. Our findings refute this assertion based on the fundamental geometry of the posterior cornea; due to its naturally concave architecture, the GL is consistently greater than the linear trephine diameter. Specifically, our data demonstrated that an 8.25 mm trephination for DMEK grafts yielded a mean GL of 8.71 mm. A similar phenomenon occurs in the convex architecture of type-1 BBs in PDEK, where the GL exceeded the BBD. This is explained by the biomechanical behavior of the tissue in BSS, where the submerged endothelial graft tends to straighten along its longitudinal axis while curling perpendicularly, effectively ‘unfolding’ its true surface area beyond the initial trephined boundary.

The metabolic integrity of the corneal endothelium is primarily maintained by the aqueous humor, which remains in intimate physiological contact with the posterior lamella. A robust correlation has been established between aqueous and systemic glucose levels [25]; notably, chronic hyperglycemia in diabetic donors is associated with advanced glycation end-products, which significantly increase the adhesive forces between the DM and the deep stroma [26]. In conventional DMEK preparation, this heightened adhesion often results in a high incidence of tissue tears and dissection failures during manual stripping techniques [27,28,29]. Consequently, some protocols even suggest the exclusion of the contralateral cornea following an unsuccessful DM removal [30].

A recent study reported successful corneal dissection for DMEK using liquid in diabetic donors [31]. In our study, which utilized air dissection, we encountered preparation failures in three out of six identified diabetic donors (50%); however, it is significant to note that all corresponding contralateral corneas were successfully processed. This observation leads us to hypothesize that the interface between the PDL and the stroma may be less susceptible to the biochemical alterations induced by chronic hyperglycemia. Due to its anterior anatomical localization and inherent impermeability, the PDL might act as a structural barrier that preserves the cleavage plane during pneumatic dissection. While our sample size is limited, these findings suggest that air dissection could offer distinct advantages for utilizing diabetic donor tissues—a possibility that warrants dedicated investigation in future prospective trials to expand the viable donor pool.

The composite graft image revealed cell loss localized mainly at fold areas, whereas the air injection site and peripheral manipulation showed minimal impact. Notably, no damage occurred adjacent to the ‘S-stamp’, a feature that typically induces endothelial loss in standard DMEK grafts. Confocal analysis confirmed robust viability post-dissection, with 2407 viable cells/mm^2^ (Hoechst) versus only 37 non-viable cells/mm^2^ (EH-1). High colocalization of CAM and Hoechst signals further validates our assessment, confirming that PDEK-related ‘scrolling’ and ‘unrolling’ maneuvers do not cause catastrophic endothelial loss. Although these qualitative findings represent a single graft and preclude broad statistical conclusions, they serve as a valuable proof of concept that aligns with our overall results, suggesting maintained endothelial integrity despite mechanical stress.

## 5. Conclusions

In conclusion, this study provides a comprehensive characterization of the digital morphometrics and scrolling dynamics of precut endothelial grafts. PDEK grafts exhibit a significantly higher CGW compared to DMEK (*p* < 0.05), primarily due to the structural retention of the PDL, and establish a clear inverse relationship between CGW and SS. Furthermore, while donor age showed a weak correlation with CGW in the DMEK group, no such correlation was observed in PDEK grafts.

## Figures and Tables

**Table 1:** Demographics data of cornea donor tissue of eye bank records.

Parameters	Value
No of donors	27
Age (y)	53.8 ± 14.5
Range	18–70
Sex	
Male	18 (66.6%)
Female	9 (33.4%)
Diabetes mellitus	4 (14.8%)
No of cornea tissues	47
Endothelial cell density (cells/mm^2^)	2.376 ± 244
Death-to-preservation time (h)	14.48 ± 8.83
Total preservation time (days)	36.56 ± 25.64
Diabetes mellitus	6 (12.7%)

**Table 2:** Scrolling Score (SS) of pre-Descemet’s endothelial keratoplasty and Descemet’s membrane endothelial keratoplasty grafts with central graft width (CGW) before and after trypan blue staining (TB-CGW).

Scroll Score	***n***	CGW	TB-CGW	Age (Years)	*p*
PDEK	24	2.35 ± 0.36	2.39 ± 0.33	50.8 ± 16.2	>0.05
SS-1	4	2.74 ± 0.30	2.81 ± 0.30	41.2 ± 26.9	0.721
SS-2	12	2.41 ± 0.26	2.41 ± 0.27	52.0 ± 13.9	0.946
SS-3	6	2.10 ± 0.38	2.19 ± 0.26	60.0 ± 11.2	0.612
SS-4	2	1.99 ± 0.50	2.11 ± 0.21	35.0 + 0.0	0.612
DMEK	17	1.95 ± 0.41	1.92 ± 0.40	55.3 ± 12.2	>0.05
SS-2	2	2.47 ± 0.14	2.46 ± 0.06	57.0 ± 0.0	0.986
SS-3	12	1.98 ± 0.34	1.97 ± 0.25	60.9 ± 4.7	0.961
SS-4	3	1.50 ± 0.34	1.34 ± 0.42	32.0 ± 8.6	0.544

**Table 3:** Pre-Descemet’s endothelial keratoplasty (PDEK) and Descemet’s membrane endothelial keratoplasty (DMEK) grafts’ mean values, standard deviation (SD) and *p* value of posterior white-to-white distance (PWTWD), big bubble diameter (BBD), graft length (GL), graft area (GA), and graft margin circularity (GMC).

	PDEK Group *n* = 24	Range	DMEK Group *n* = 17	Range	*p*
PWTWD	11.66 ± 0.38	(11.07–12.41)	11.75 ± 0.44	(11.07–12.56)	0.496
BBD	7.40 ± 0.44	(6.48–8.37)	10.40 ± 0.46	(9.11–10.81)	<0.001
GL	7.91 ± 0.41	(7.10–8.69)	8.71 ± 0.51	(7.71–9.72)	<0.001
GA	50.76 ± 7.12	(33.20–66.19)	60.99 ± 9.2	(47.86–78.18)	<0.001
GMC	0.81 ± 0.06	(0.69–0.91)	0.89 ± 0.02	(0.82–0.92)	<0.001

## Data Availability

The datasets generated and/or analyzed during this study are available from the corresponding author upon reasonable request.

## Abbreviations

The following abbreviations were used in this manuscript:

DMEK	Descemet membrane endothelial keratoplasty
PDEK	Pre-Descemet endothelial keratoplasty
CGW	Central graft width
TB	Trypan blue
TB-CGW	Central graft width after trypan blue
ECD	Endothelial cell density
DPT	Death-to-preservation time
TPT	Total preservation time
PT	Procedure time
AIV	Air-injected volume
PWTWD	Posterior white-to-white distance
BBD	Big bubble diameter
GL	Graft length
GA	Graft area
GMC	Graft margin circularity
EH-1	Ethidium-homodimer
CAM	Calcein AM
